# Circulating Tumor DNA to Interrogate the Safety of Letrozole-Associated Controlled Ovarian Stimulation for Fertility Preservation in Breast Cancer Patients

**DOI:** 10.3389/fonc.2021.686625

**Published:** 2021-08-03

**Authors:** Françoise Rothé, Matteo Lambertini, Oranite Goldrat, Marion Maetens, Yacine Bareche, Jeremy Blanc, Ghizlane Rouas, Denis Larsimont, Christos Sotiriou, Michail Ignatiadis, Isabelle Demeestere

**Affiliations:** ^1^Breast Cancer Translational Research Laboratory J.C. Heuson, Institut Jules Bordet, Université Libre de Bruxelles (ULB), U-CRC, Bruxelles, Belgium; ^2^Department of Internal Medicine and Medical Specialties (DiMI), School of Medicine, University of Genova, Genova, Italy; ^3^Department of Medical Oncology, U.O.C. Clinica di Oncologia Medica, IRCCS Ospedale Policlinico San Martino, Genova, Italy; ^4^Fertility Clinic, CUB-Erasme Hospital, Brussels, Belgium; ^5^KU Leuven, Department of Oncology, Laboratory for Translational Breast Cancer Research, Leuven, Belgium; ^6^Pathology Department, Institut Jules Bordet, Brussels, Belgium; ^7^Medical Oncology Department, Institut Jules Bordet, Université Libre de Bruxelles, Bruxelles, Belgium; ^8^Research Laboratory on Human Reproduction, Université Libre de Bruxelles, Brussels, Belgium

**Keywords:** breast cancer, fertility preservation, letrozole, ovarian stimulation, circulating tumor DNA

## Abstract

**Background:**

Current fertility preservation strategies for young breast cancer patients planning a future motherhood include the association of controlled ovarian stimulation with the aromatase inhibitor letrozole (let-COS) to harvest mature oocytes while maintaining low estradiol levels. Despite this is a widely adopted protocol, the safety of let-COS on breast cancer outcomes has been poorly investigated and its use remains off-label. We assessed the safety of let-COS in breast cancer patients using circulating tumor DNA (ctDNA) as a surrogate biomarker of disease recurrence.

**Methods:**

BROVALE is an interventional non-randomized prospective study designed to evaluate the efficacy and safety of let-COS for fertility preservation in early breast cancer patients before starting (neo)adjuvant chemotherapy. Letrozole was administered throughout the COS cycle, until ovulation triggering. Safety was a secondary endpoint. Data on oncological outcomes were collected during the follow-up as well as plasma and whole blood for evaluation of ctDNA levels at the time of enrollment (i.e. before starting let-COS) and oocyte retrieval (i.e. 48 hours after the last administration of letrozole). Targeted gene sequencing on the primary tumor samples was performed to identify specific mutations used for ctDNA analysis by digital PCR. DNA extracted from whole blood samples was used to discriminate between somatic and germline mutations.

**Results:**

From April 2014 to May 2017, 29 young early breast cancer patients enrolled in the BROVALE study who had available tissue samples participated to the ctDNA substudy. Among them, 15 had at least one validated somatic mutation. ctDNA was undetectable neither before nor after let-COS in 9 of them. Six patients had detectable ctDNA in the plasma samples collected before Let-COS. No change in ctDNA level after let-COS was observed in 3 patients and the level decreased (fold-change ≤ 0.5) in two women. One patient experienced an increased (fold-change ≥ 2) in ctDNA level but without disease relapse 34 months after diagnosis.

**Conclusions:**

No increase in ctDNA level was observed in 93% (14/15) of the patients receiving let-COS supporting its use as a safe strategy for young women with early breast cancer interested in fertility preservation before chemotherapy.

## Introduction

Recent advances in screening procedures and anticancer treatments have markedly improved survival in young early breast cancer patients (1). The majority of young women with newly diagnosed early breast cancer are candidates to receive neoadjuvant or adjuvant chemotherapy including gonadotoxic drugs that might severely impact their reproductive function and future fertility (2, 3). Therefore, oncofertility counseling is currently mandatory in all patients diagnosed during their reproductive years and, for women planning a future motherhood, fertility preservation before starting chemotherapy is standard of care (4–6).

Oocyte and/or embryo cryopreservation is currently the first strategy for fertility preservation to be offered to young early breast cancer patients (7). The standard approach to collect a maximum number of mature oocytes includes 10-15 days of controlled ovarian stimulation (COS) with gonadotropins using a gonadotropin-releasing hormone (GnRH) antagonist protocol to avoid premature spontaneous luteinizing-hormone (LH) peak (8). As this protocol is associated with a supraphysiological raise in estradiol levels, concerns have been raised on its potential detrimental prognostic effect in hormone-sensitive cancer such as breast cancer (9, 10). The co-administration of an aromatase inhibitor (letrozole) during COS allows to harvest several mature oocytes while maintaining low estradiol levels (11–13). A recent meta-analysis of 11 studies comparing standard COS with protocols including the administration of letrozole confirmed a similar efficacy in terms of oocyte yield, maturation and fertilization rates, but with significantly reduced estradiol levels when letrozole is included in the COS protocol (14). Despite this is a widely adopted protocol, the safety of letrozole-associated COS (let-COS) on breast cancer outcomes has been poorly investigated and its use is currently off-label in this indication.

Liquid biopsy evaluating the presence of circulating tumor DNA (ctDNA) is widely used as a minimally invasive tool offering a wide range of clinical applications (15). Among them, the detection of ctDNA during follow-up has been shown to be associated with a high risk of disease relapse in patients with early breast cancer (16–19).

In this study, we aimed to explore the safety of let-COS for oocyte and/or embryo cryopreservation in a prospective cohort of young women with early breast cancer who preserved their fertility before chemotherapy. For this purpose, in addition to oncological outcomes, we explored potential changes in ctDNA levels before and after let-COS as a possible surrogate measure of tumor development and predictor of disease relapse.

## Methods

### Patient Population

BReast cancer OVAry LEtrozole (BROVALE) (NCT02661932) is an interventional non-randomized prospective study designed to evaluate the efficiency and safety of let-COS for fertility preservation in young women with early breast cancer. Details of the study have been previously reported (12). The present biomarker analysis addressed one of the planned secondary endpoints of the study focusing on the safety of let-COS. For this purpose, the changes in ctDNA levels before and after let-COS as well as oncological outcomes were assessed.

In BROVALE, standard or random start COS protocol using gonadotropins (150 to 300 IU/day) and GnRH antagonist (0.25mg/d from day 6, or when follicles reached 14 mm) was applied in all patients. GnRH agonist or human chorionic gonadotropin (hCG) were used for triggering when at least two follicles exceed 18mm and transvaginal ultrasound-guided oocyte retrieval occurred 36 hours later. Letrozole (5mg/day per os) was administered throughout the COS cycle, starting one day before or concomitantly with gonadotropins until ovulation triggering as previously described (12).

The Ethic Committee of Erasme Hospital approved the study. Informed consent was obtained from all participants before study inclusion.

### Study Procedures

Whole blood samples for genomic DNA preparation were collected in EDTA tubes at the time of enrollment (i.e. before let-COS) and at oocyte retrieval (i.e. 36 hours after last administration of letrozole). Plasma and whole blood were immediately stored at -80°c until DNA extraction. Formalin fixed paraffin embedded (FFPE) tumor samples were collected from participating patients.

Plasma cell-free DNA (cfDNA) was extracted using the QIAamp circulating nucleic acid kit (Qiagen). Genomic DNA was extracted from whole blood samples using the Qiagen DNeasy Blood & Tissue Kit to discriminate somatic from germline mutations. DNA from primary tumor samples (FFPE) was extracted using the Qiagen QIAamp DNA FFPE tissue kit.

Somatic mutations were identified from primary tumor samples by targeted gene sequencing using the Truseq Amplicon Cancer 48-gene Panel (Illumina, reference FC-130-1008). Sequence reads from the tumor and normal samples were aligned against the human genome reference version hg19/GRCh37 using the BWA (v.0.7.15) aligner with default parameter settings. In order to correct for mapping errors made by BWA around indels, a local realignment step was performed using IndelRealigner from the GATK (v.4.0.3.0) suite. When matched normal genomic DNA was available, somatic mutation calling was performed with two distinct variant callers, Manta (v.1.3.2)/Strelka (v.2.9.2) and Mutect 2 (v.4.0.3.0), using default parameters. When matched normal genomic DNA was not available, mutation calling was performed with two distinct variant callers, Pisces (v.5.1.6.54) & Mutect 2 (v.4.0.3.0), using default tumor mode only. Somatic mutations were annotated using ANNOVAR. Mutations were then filtered by selecting only exonic, non-synonymous single nucleotide variant (SNV) with a variant allele frequency (VAF) ≥ 8% and a coverage ≥ 1000 reads. Only known COSMIC (v.81) mutations with a frequency lower than 1% in the ExAC (v.0.3.1) database were used in further analysis.

The presence of plasma ctDNA was evaluated using the highly sensitive and precise digital PCR, a refined method of the conventional polymerase chain reaction (PCR). In particular, patient-specific droplet digital PCR (ddPCR) assays (Biorad PrimePCR ddPCR Mutation Assay or custom Assay) were used to detect the mutations identified in the tumor samples, with a single mutation being selected for each patient as previously reported (20).

## Results

Between April 2014 and May 2017, 31 early breast cancer patients with available tissue samples participated in the BROVALE ctDNA study. Two patients were excluded from further analysis due to low tumor DNA quantity (<50 ng; **Supplementary Figure 1**). All included patients underwent let-COS for fertility preservation before starting (neo)adjuvant chemotherapy. Out of 29 patients included in the present analysis, 12 (41.4%) had estrogen receptor (ER)-positive/HER2-negative tumors, 12 (41.4%) HER2-positive disease and 5 (17.2%) triple-negative breast cancer. Patients’ and oncological characteristics are summarized in **Table 1**.

**Table 1:** Patients and tumor characteristics (n=29).

	All patients (n = 29)	Patients without mutation (n = 14)	Patients with mutation (n = 15)	P value*
**Age, IQR**	31 (29-35)	31.6 (28.5-34.8)	32.4 (30-35)	0.57
**Clinical setting**				
Adjuvant	15 (51.7)	9 (64.3)	6 (40.0)	0.35
Neoadjuvant	14 (48.3)	5 (35.7)	9 (60.0)	
**Tumor size**				
0.1-5 cm	26 (89.7)	12 (85.7)	14 (93.3)	0.95
>5 cm	3 (10.3)	2 (14.3)	1 (6.7)	
**Nodal status**				
Negative	20 (69.0)	9 (64.3)	11 (73.3)	0.9
Positive	9 (31.0)	5 (35.7)	4 (26.7)	
**Grade**				
I/II	11 (37.9)	6 (42.9)	5 (33.3)	1
III	18 (62.1)	8 (57.1)	10 (66.7)	1
**Ki67%, IQR**	54.3 (20-80)	50.3 (20-84)	58 (35-78)	0.66
**Estrogen receptor status**				
Negative	8 (27.6)	3 (21.4)	5 (33.3)	0.88
Positive	21 (72.4)	11 (78.6)	10 (66.7)	
**Progesterone receptor status**				
Negative	12 (41.4)	5 (35.7)	7 (46.7)	0.76
Positive	17 (58.6)	9 (64.3)	8 (53.3)	
**HER2 status**				
Negative	17 (58.6)	8 (57.1)	9 (60.0)	0.83
Positive	12 (41.4)	6 (42.9)	6 (40.0)	
**Parity**				
Parous	5 (17.2)	2 (14.3	3 (20.0)	1
Nulliparous	24 (82.8)	12 (85.7)	12 (80.0)	

^*^Wilcoxon rank sum test for continuous variables and Fisher test for categorical variables, for the comparison between patients with and without mutation.

IQR, interquartile range.

**Figure 1:**
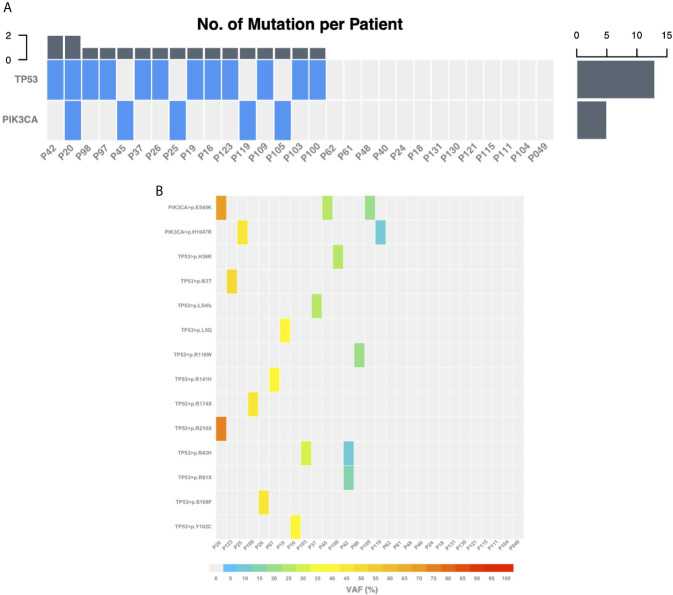
Somatic mutations identified using targeted gene sequencing on the primary tumor samples. **(A)** Heatmap of genes for which at least one mutation was indexed across the 29 patients. **(B)** Heatmap of the variant allele frequency for each specific mutation indexed across the 29 patients. VAF, variant allele frequency.

**Figure 2:**
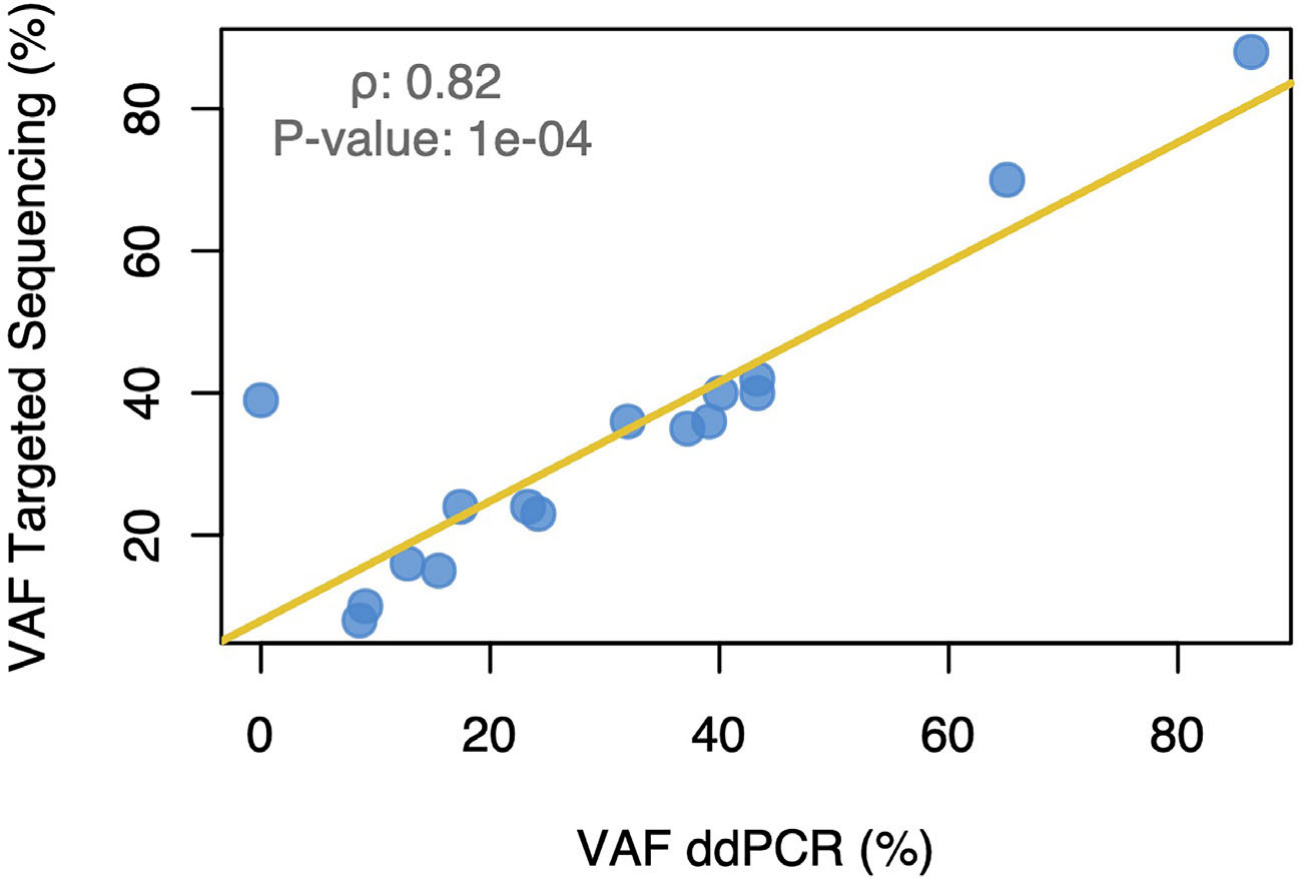
Correlation between the variant allele frequency of the somatic mutations identified using targeted gene sequencing and droplet digital PCR. VAF, variant allele frequency; ddPCR, droplet digital PCR.

Targeted gene sequencing was performed on primary tumor samples of the 29 included patients in order to identify somatic mutations for subsequent plasma ctDNA detection. Sixteen (55%) tumor samples presented at least 1 somatic mutation either in *TP53* (44.8%) or *PIK3CA* (17.2%) genes. No mutations could be identified in the other interrogated genes. A single mutation was selected for each of the 16 patients, being *TP53* and *PIK3CA* mutations in 11 and 5 patients, respectively (**Figures 1A, B**). Fifteen of them (93.8%) were further validated using highly sensitive patient-specific mutation ddPCR assays with a high concordance being observed in the variant allelic frequency (VAF) between targeted gene sequencing and ddPCR (**Figure 2**).

For these 15 patients, the median duration of the stimulation was 9 days (range 5-14) and median estradiol peak reached 291pg/ml (range 55-928). A median of 6 mature oocytes were collected (range 1-21) (**Table 2**).

**Table 2:** Characteristics of the COS cycles.

Patients ID	AMH (ng/ml)	Total doses of gonadotropins (IU)	COS duration (days)	E2 peak at triggering (pg/ml)	Triggering	Oocytes yield (N)
P20	0.42	3300	13	55	hCG	2
P103	1.9	3950	14	238	GnRHa	6
P37	6.1	1338	8	469	GnRHa	21
P16	0.1	2775	11	95	hCG	3
P26	0.54	450	5	65	hCG	1
P123	3.9	2250	9	472	GnRHa	15
P19	0.67	1700	10	487	hCG	7
P25	0.24	2250	9	291	hCG	4
P42	0.44	2700	9	92	GnRHa	3
P45	–	2400	8	133	GnRHa	2
P98	1.7	2250	9	468	GnRHa	10
P100	4.5	3500	13	747	GnRHa	10
P105	2.9	1800	8	615	GnRHa	5
P109	5.7	2200	11	928	GnRHa	16
P119	2.2	2025	9	291	GnRHa	11

AMH, Anti-Müllerian Hormone; COS, Controlled Ovarian Stimulation; hCG, human Chorionic Gonadotropin; GnRHa, Gonadotropin Releasing Hormone Agonist.

**Figure 3:**
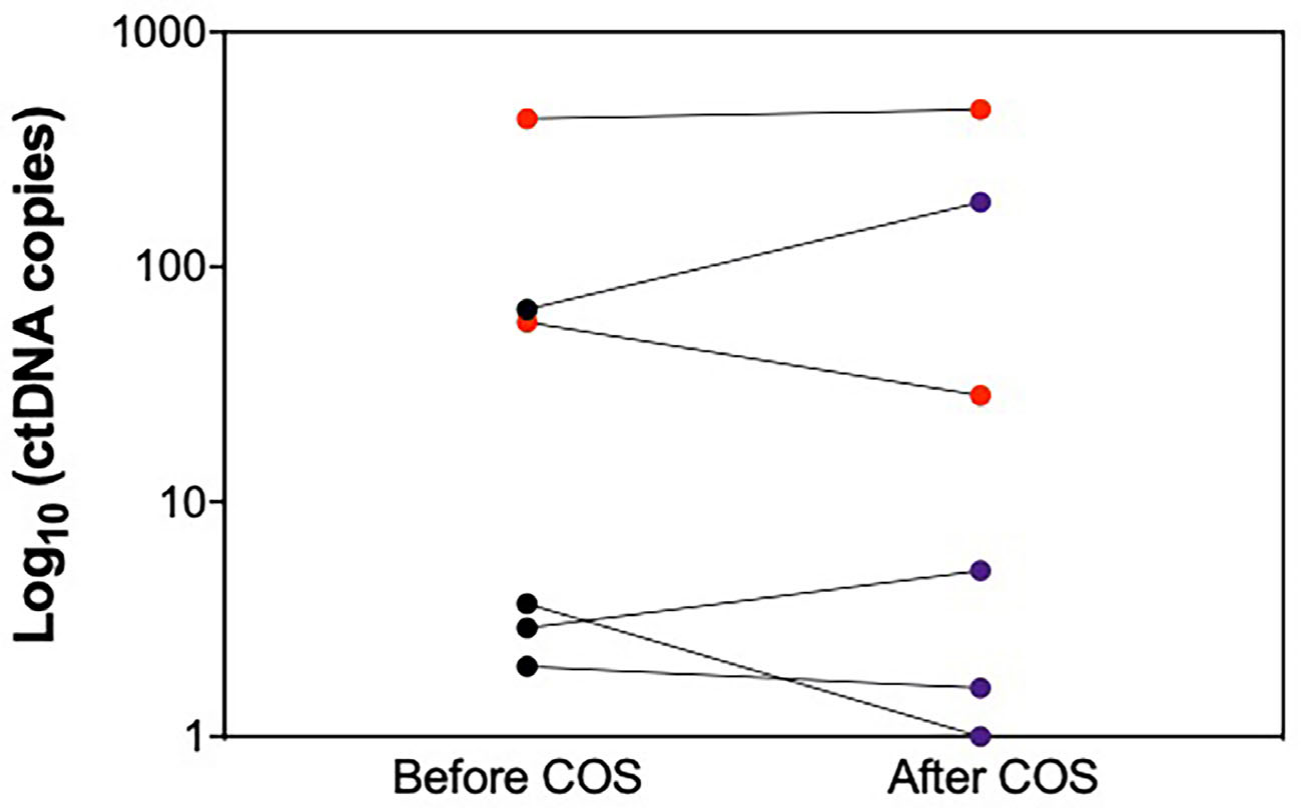
Changes in circulating tumor DNA levels between the time of enrollment (i.e. before starting letrozole-associated controlled ovarian stimulation) and oocyte retrieval (i.e. 48 hours after the last administration of letrozole). Red dots = disease relapse during oncologic follow-up. Violet = no disease relapse during oncologic follow-up. ctDNA, circulating tumor DNA; COS, controlled ovarian stimulation.

The presence of ctDNA was assessed in the plasma samples collected before and after Let-COS using ddPCR. In 9 out of 15 patients, ctDNA was not detectable before nor after let-COS. None of them had disease relapse during follow-up (**Table 3**). Six patients had detectable ctDNA in the plasma samples collected before Let-COS (**Figure 3**). An increase in ctDNA level after let-COS (fold-change ≥ 2) was observed in only one patient without disease relapse at the last follow-up visit 34 months after breast cancer diagnosis (P123). On the contrary, 3 patients had no change in ctDNA level after let-COS (P103-P16-P26), one of whom developed disease-relapse after 13 months of follow-up and died (P26). This patient was diagnosed with triple-negative breast cancer (T2N2) and had the highest average number of mutated copies in the plasma before and after the procedure (427.03 and 467.67 ctDNA copies/ml, respectively). Other 2 patients (P20-P37) had a decrease in ctDNA level after let-COS (fold-change ≤ 0.5), one of whom developed disease-relapse (P37) (**Table 3**).

**Table 3:** Characteristics of the patients with identified mutations, targeted gene sequencing and droplet digital PCR results and changes in circulating tumor DNA before and after controlled ovarian stimulation (total n = 15).

Patients characteristics					Tumor characteristics									ctDNA before COS		ctDNA after COS		
Patient ID	Age	Relapse	Alive	DFS follow-up (month)	Subtype	T	N	Ki67%	Gene	Mutation	ddPCR assay type	Primary VAF NGS %	Primary VAF ddPCR %	Plasma VAF %	Copies mutated average/ml plasma	Plasma VAF %	Copies mutated average/ml plasma
P20	27	No	Yes	68	ER+/PR+/HER2+	2	0	75	PIK3CA	pE545K	PrimePCR ddPCR Mutation Assay	70	65.1	0.75	3.68	0	0
P103	34	No	Yes	40	TNBC	2	–	70	TP53	p.R175H/R43H	PrimePCR ddPCR Mutation Assay	35	37.2	0.27	1.99	0.14	1.61
P37	28	Yes	Yes	57	ER+/PR-/HER2-	2	0	75	TP53	p.L54fs	PrimePCR ddPCR Custom Assay	24	17.4	1.79	58.27	1.95	28.37
P16	35	No	Yes	65	TNBC	2	0	90	TP53	p.Y102C/Y234C	PrimePCR ddPCR Custom Assay	36	39.1	0.22	2.91	0.26	5.10
P26	35	Yes	No	13	TNBC	2	2	95	TP53	p.S109F/S241F	PrimePCR ddPCR Custom Assay	40	40.1	22.5	427.03	28.75	467.67
P123	34	No	Yes	34	ER-/PR-/HER2+	2	0	90	TP53	p.I63T	PrimePCR ddPCR Mutation Assay	88	86.4	9.05	65.93	14.35	188.60
P19	34	No	Yes	56	ER+/PR+/HER2+	1	1	80	TP53	p.L5Q	PrimePCR ddPCR Custom Assay	36	32	0	0	0	0
P25	35	No	Yes	27	ER+/PR+/HER2-	1	0	35	PIK3CA	p.H1047R	PrimePCR ddPCR Mutation Assay	42	43.3	0	0	0	0
P42	35	No	Yes	13	ER+/PR+/HER2-	1	0	64	TP53	p.R175H/R43H	PrimePCR ddPCR Mutation Assay	10	9.1	0	0	0.045	0.61
P45	36	No	Yes	46	ER+/PR+/HER2-	2	0	60	PIK3CA	pE545K	PrimePCR ddPCR Mutation Assay	24	23.3	0.07	0.69	0	0
P98	24	No	Yes	50	ER+/PR-/HER2+	3	0	10	TP53	p.R248W/R116W	PrimePCR ddPCR Mutation Assay	16	12.8	0	0	0.0095	0.54
P100	32	No	Yes	41	ER-/PR-/HER2+	1	1	60	TP53	p.H36R	PrimePCR ddPCR Custom Assay	23	24.2	0	0	0	0
P105	31	No	Yes	37	ER+/PR+/HER2-	2	0	16	PIK3CA	pE545K	PrimePCR ddPCR Mutation Assay	15	15.5	0	0	0.13	0.46
P109	29	No	Yes	30	ER+/PR+/HER2+	1	–	15	TP53	p.R174X	PrimePCR ddPCR Mutation Assay	40	43.3	0.014	2.76	0	0
P119	37	No	Yes	15	ER+/PR+/HER2-	2	0	35	PIK3CA	p.H1047R	PrimePCR ddPCR Mutation Assay	8	8.6	0	0	0	0

ctDNA, circulating tumor DNA; COS, controlled ovarian stimulation; NGS, targeted gene sequencing; ddPCR, droplet digital PCR; DFS, disease-free survival; ER, estrogen receptor; PR, progesterone receptor; TNBC, Triple-Negative Breast cancer; VAF, variant allele frequency.

## Discussion

In young women with early breast cancer interested in preserving fertility before starting neoadjuvant or adjuvant chemotherapy, oocyte and/or embryo cryopreservation following Let-COS protocol is widely adopted and recommended (21–23). However, the safety of this approach relies mainly on one single-center prospective non-randomized study showing no difference in risk of recurrence between 120 breast cancer patients who performed Let-COS for oocyte and/or embryo cryopreservation and a control group of 217 patients who did not preserve their fertility before starting chemotherapy (13). In a recent large prospective multicenter Swedish study including 380 women with breast cancer who underwent COS for fertility preservation between 1995 and 2017, the 5-year survival proportion was similar compared to breast cancer patients who did not perform COS (24). In this study, Let-COS was offered to only 59% of the patients. Moreover, oncological characteristics of the population were not reported, leading to important potential biases in the survival analysis (24).

Therefore, defining the safety of performing COS for oocyte and/or embryo cryopreservation in breast cancer patients remains a clinical research priority (25). As shown in a recent survey involving breast cancer specialists, more than one third of them are concerned about the potential detrimental prognostic effect of COS in patients with breast cancer (10).

To our knowledge, this biomarker analysis is the first study addressing the safety of performing let-COS for fertility preservation in young breast cancer patients using ctDNA as a surrogate biomarker of disease recurrence. Indeed, among the wide range of clinical applications of this sensitive minimally invasive tool, molecular relapse detection is one of the most promising (16–19).

We first performed targeted gene sequencing in the primary tumors in order to identify the somatic mutations to be assessed for ctDNA detection in the plasma samples. Mutations were only present in *TP53* and *PIK3CA* genes that are known to be the two most frequently mutated genes in breast cancer (26). Notably, 15 out of the 16 mutations identified in the primary tumors were further validated using ddPCR. In our study, ctDNA was detected in 40% of the plasma samples before let-COS and the initiation of chemotherapy. This is similar to previous studies reporting a detection rate of approximately 50% in patients with newly diagnosed early breast cancer irrespective of molecular subtype and prior to any treatment (27).

Reassuringly, let-COS did not induce the emergence of ctDNA in these patients, although the majority of patients had hormone receptor-positive disease and supraphysiological estradiol levels (>500pg/ml) were reached in a third of them. In our study, ctDNA was detected in 6 patients at enrollment but increased in only one of them. Importantly, no negative effect on her oncological outcomes was observed. Notably, the patient exhibiting the highest ctDNA level at both time-points relapsed shortly after entering the study and died. She was affected by triple-negative breast cancer and had the shortest stimulation duration characterized by very low estradiol levels during COS. On the contrary, all patients with undetectable or very low ctDNA levels remained disease-free at the time of the last follow-up. The observation that there was no increase in ctDNA levels in the majority of the patients indirectly supports the lack of potential detrimental prognostic effect of a short-course of hormonal manipulation with let-COS in young women with early breast cancer before exposure to chemotherapy.

In terms of study limitations, this biomarker analysis has a relatively limited sample size. Formal statistical calculations could not be performed. Moreover, despite promising, to date the role of ctDNA as a tool for disease monitoring in patients with early breast cancer remains experimental without direct clinical application yet. However, importantly, this analysis was conducted within an interventional non-randomized prospective study and all biological samples were prospectively collected.

In conclusion, this biomarker analysis of the BROVALE study showed no increase in ctDNA levels in 93% of young women with early breast cancer who received let-COS for oocyte and/or embryo cryopreservation as a strategy to preserve fertility before starting neoadjuvant or adjuvant chemotherapy. These data indirectly support the use of this strategy as a safe approach in young early breast cancer patients interested in fertility preservation before chemotherapy initiation. Further validation of these findings in a large prospective clinical trial is warranted.

## Data Availability Statement

The original contributions presented in the study are included in the article/**Supplementary Material**, further inquiries can be directed to the corresponding author.

## Ethics Statement

This study involving human participants was reviewed and approved by Erasme Hospital. The patients/participants provided their written informed consent to participate in this study.

## Author Contributions 

FR, ML, MI, and ID contributed to the conception and design of the study. OG and ID contributed to patients’ enrollment in the BROVALE study and sample collection. FR, ML, MM, YB, JB, GR, DL, CS, and MI contributed to sample storage, processing, analysis and interpretation. The results were interpreted by FR, ML, MI, and ID. The initial manuscript was drafted by FR, ML, MI, and ID. All authors contributed to the article and approved the submitted version.

## Funding

This study received partial financial support from the Belgian Fund for Scientific Research (FNRS)-Operation Télévie (grant number: 7452815F), Fonds Erasme (no grant number), and by an ESMO Translational Research Fellowship grant (no grant number). ML is supported by the Italian Ministry of Health - 5 x 1000 funds 2017 (no grant number) and the Italian Association for Cancer Research (AIRC; grant number MFAG 2020 ID 24698) for pursuing in his research efforts in the field of oncofertility.

## Conflict of Interest

ML acted as a consultant for Roche, AstraZeneca, Novartis, and Lilly, and received honoraria from Roche, Lilly, Novartis, Pfizer, Sandoz, and Takeda outside the submitted work. MI acted as a consultant for Novartis, and Seattle Genetics, and his Institution received research grants from Roche, Pfizer and Natera Inc. ID acted as a consultant for Roche and received speaker’s fees from Novartis.

The remaining authors declare that the research was conducted in the absence of any commercial or financial relationships that could be construed as a potential conflict of interest.

The reviewer FS declared a past co-authorship with one of the authors ML to the handling editor.

## Publisher’s Note

All claims expressed in this article are solely those of the authors and do not necessarily represent those of their affiliated organizations, or those of the publisher, the editors and the reviewers. Any product that may be evaluated in this article, or claim that may be made by its manufacturer, is not guaranteed or endorsed by the publisher.
