# Supplementary material for: Circulating Tumor DNA to Interrogate the Safety of Letrozole-Associated Controlled Ovarian Stimulation for Fertility Preservation in Breast Cancer Patients
Source: Front Oncol. 2021 Aug 3;11:686625. doi: 10.3389/fonc.2021.686625 (PMC8370091; doi:10.3389/fonc.2021.686625)
Supplement: Supplementary file 1 [file DataSheet_1.docx]

**Supplementary Figure 1**. Study Flow
